# Specific histamine regulating activity of surface‐modified yeast vacuoles by histamine‐ binding protein and its immune‐enhancing effect

**DOI:** 10.1111/1751-7915.14116

**Published:** 2022-08-10

**Authors:** Hyeweon Jang, Yang‐Hoon Kim, Jiho Min

**Affiliations:** ^1^ Graduate School of Semiconductor and Chemical Engineering Jeonbuk National University Jeonju‐si Korea; ^2^ School of Biological Sciences Chungbuk National University Cheongju South Korea

## Abstract

We aimed to develop a biocompatible material that could enhance weakened immunity and control histamine in vivo. Histamine‐binding protein (HBP) vacuoles have a mechanism of action that directly binds to the histamine molecule. It is designed to eliminate the side effects of antihistamine caused by binding to other receptors. HBP vacuoles were designed to produce a material that was biocompatible, and could enhance immunity. First, a recombinant vector was designed so that HBP was located on the vacuole surface, and expressed towards the cytoplasm. The vector was transformed into yeast, and protein expression was induced. Then, the vacuole was isolated by centrifugation to complete HBP vacuoles. Cytotoxicity test was conducted for application to RAW 264.7 cells. In addition, immune enhancement reaction and histamine inhibition were confirmed through phagocytosis assay and histamine ELISA. RAW 264.7 cells were pre‐treated with HBP vacuoles to confirm the immune enhancement of HBP vacuoles. As a result, it was confirmed that the immunostimulatory effect of the vacuole was increased in a concentration‐dependent manner. In addition, the reduction of histamine was confirmed by treating the HBP vacuoles. As a result, HBP vacuoles reduced the histamine secreted from RAW 264.7 cells by about 75%.

## Graphical abstract

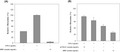

## INTRODUCTION

Histamine (HA) is a bioamine that acts as a mediator of inflammatory and immune responses in the human body. HA is mainly synthesized by mast cells and basophils, stored in granules, and secreted by external stimuli (Dy & Schneider, 2004). Also, HA is synthesized in small amounts by T cells, dendritic cells, epithelial cells and macrophages (Szeberényi et al., 2001). HA expands capillaries, facilitates the movement of various immune cells through the expanded blood vessels, triggers an inflammatory response and helps control the invasion of foreign substances and decompose harmful substances (MacDermot, 1927).

Atopic dermatitis (AD) is a chronic skin disease that afflicts all generations from children to the elderly, and causes an excessive inflammatory reaction due to damage to the skin barrier and the invasion of external antigenic substances and pathogens, resulting in pruritus, skin redness, lichenification and skin infections (Ellis et al., 2012; Schmitt et al., 2008). AD patients have a higher number of mast cells than normal people who do not, and the level of HA is maintained high. Therefore, even the slightest stimulation causes an inflammatory response and itching (de Luca et al., 1995). Anti‐HAs are prescribed to relieve this itching (Klein & Clark, 1999). Anti‐HAs inhibit the action of HA by binding to HA receptors, but also bind to other receptors to show side effects. Because of these side effects, such as drowsiness, constipation and blurred vision, there is a need for a drug that inhibits HA in a different direction from anti‐HAs. Despite these side effects, we have limited choices, as to date there are no suitable substitutes (Hindmarch & Shamsi, 1999).

Blood‐sucking mites, especially *Rhipicephalus appendiculatus*, secrete histamine‐binding proteins (HBPs) into the wounds of host animals for successful feeding. The secreted HBPs inhibit the action of HA, which causes an inflammatory response. HBPs also aid the adhesion of ticks and reproductive activity (Paesen et al., 2000).

Yeast is a familiar microorganism, because it is widely used as a food and bio‐product. In addition, it is used as a microorganism that is genetically engineered to produce useful materials; because it is easy to mass‐produce, it is widely used (Halász & Lásztity, 2017; Johnson & Echavarri‐Erasun, 2011).Yeast vacuole is the most acidic among organelles, and is effective in protein degradation. It is also a digestive organelle that plays a role in cellular response and detoxification to external stimuli. Also, it has antibacterial and anticancer effects, and has the effect of recovering a weakened immune system by stimulating immunity in vitro (Lee et al., 2021; Yoon et al., 2016, 2017). In addition, it is good for bioengineered materials, and can be used as a drug delivery system (Armstrong, 2010).

This study is a result of the development of a biotechnological product that is capable of regulating the behaviour of HA molecules, using genetic recombination techniques and confirmation of the safety of new materials. The agents used in this study are bioengineered materials that bind to HA and control the behaviour of HA. In addition, AD, the disease targeted in this study, is a chronic inflammatory disease, so the agents that enhance immunity are needed. Therefore, this effect could be expected by using yeast vacuoles with the advantage of immune‐enhancing effect.

## EXPERIMENTAL PROCEDURES

### Construction of recombinant plasmid

The gene sequence of HBP2 (Female‐specific Histamine‐Binding Protein 2, *Rhipicephalus appendiculatus*, GenBank accession no. U96081) was obtained by requesting gene synthesis from Bioneer (Daejeon, Korea). The gene sequence of permuted‐Vacuolar Membrane ATPase 11 (pVMA11) is a modification of VMA11 (GenBank accession no. NC_001148), such that the N‐terminus and C‐terminus are directed to cytoplasm.

The plasmid was constructed in the order of Signal sequence (SS, 117 bp), Histamine‐binding protein 2 (HBP2, 513 bp), Green fluorescence protein (GFP, 714 bp), pVMA11 (391 bp), and His tag (18 bp), and it was based on pYES2 vector (Invitrogen). The vector was called HBP vector. As controls, pVMA11 vector was constructed with pYES2::SS::GFP::pVMA11::His tag, and mock vector consisting of pYES2 vector was used.

### Construction of plasmid DNA pYES2::SS::HBP2::GFP::pVMA11::His tag

The vector gene was used based on the pVMA11 vector (Kim et al., 2019), and GFP gene was inserted between pVMA11 Signal sequence and pVMA11 gene. Then, the vector was completed by ligating HBP2 gene in front of GFP gene, and amplified through *Escherichia coli* (*E. coli*) DH5α.

### Transformation of the complete plasmid to *S. cerevisiae*

The completed vector was transformed into yeast by Lithium acetate method (Schiestl & Gietz, 1989), and *Saccharomyces cerevisiae* 2805 strain (ATCC 208280) was used for yeast transformation. *S. cerevisiae* was cultured in Yeast Extract–Peptone–Dextrose (YPD) medium (1% yeast extract, 2% peptone and 2% glucose) up to OD600 of (0.5–0.7) at 30 °C. Cells was centrifuged, and washed with Lithium acetate–Tris–EDTA (LiOAc–TE) solution (10 mM of Tris–HCl, 1 mM of EDTA, 0.1 M of Lithium acetate, pH 7.5). The cells were resuspended in LiOAc–TE solution, and incubated for 20 min at 30°C. The cells were mixed with plasmid, and incubated for 10 minutes at 30°C. Polyethylene glycol (PEG) mix was added to the cells (40% PEG, 10 mM of Tris–HCl, 1 mM of EDTA, 0.1 M of lithium acetate, pH 7.5), and the cells were incubated for 30 min at 30°C. Heat shock was performed to 42°C to allow the plasmid to enter the yeast. Supernatant was removed by centrifugation, and the cells were resuspended in synthetic dextrose (SD) medium (0.67% yeast nitrogen base, 0.5% casamino acid and 2% d‐glucose). Finally, the cells were spread on SD solid medium (0.67% yeast nitrogen base, 0.5% casamino acid, 2% d‐glucose and 2% agar).

### Isolation of HBP vacuoles

Recombinant yeast was cultured in SD medium for 24 hours (h) at 30 °C, and protein expression induced with 2% galactose using galactose promoter for 20 h. The cells were then collected, and washed with distilled water (DW). To separate vacuoles, the cells was incubated with Tris‐SO_4_ buffer (0.1 M Tris‐SO_4_, 10 mM DTT) at a volume ratio of 1:5 at 30°C. The supernatant was removed. Glass beads of the same mass as the cells were added to the cells, breaking buffer (20 mM of Tri–HCl, pH 7.4, 0.6 M of Sorbitol, 1 mM of PMSF) was added at a volume ratio of 1:4, and the cells were vortexed to disrupt them. The vacuoles were separated by centrifugation. To measure the concentration of the vacuole, the vacuoles were lysed with lysis buffer (0.1 NP‐40, 5 mM DTT, 0.1 mM PMSF) by vortex, and the proteins in the supernatant were estimated by Quick Start Bradford 1X Dye reagent (Bio‐rad). We named the vacuoles HBP vacuoles, and the vacuoles used as controls were named mock vacuoles and pVMA11 vacuoles.

### Western blot

HBP vacuoles were lysed, and the protein amount was estimated by Bradford protein assay. The samples were run on a 10% SDS–PAGE, transferred to nitrocellulose membranes and blocked with 5% skim milk in T–TBS. The membranes for HBP vacuoles were incubated overnight at 4 °C with Anti‐6X His tag antibody (abcam, UK, 1:1000 dilution). The blots were washed and incubated at room temperature for 1 h with Gt anti–Ms IgG (H + L) secondary antibodies (Invitrogen, 1: 5000 dilution). The membranes were washed and reacted with ECL STAR (DYNE, Korea). Immunofluorescent blots were developed by X‐ray film (AGFA).

### Animal cells culture

The murine macrophage RAW 264.7 cells were purchased from the Korean cell line bank (KCLB), and were cultured in Dulbecco's Modified Eagle's Medium (DEME, GIBCO), supplemented with 10% Foetal Bovine Serum (FBS, GIBCO), 100 U/ml penicillin and 100 μg/ml streptomycin in a humidified 5% CO_2_ atmosphere at 37°C.

### Cell viability

Cytotoxicity tests were performed to find the appropriate treatment concentration of HBP vacuoles. RAW 264.7 cells were adjusted to 5 × 10^4^/ml, cultured for 24 h and starved for 12 h. RAW 264.7 cells were treated with HBP vacuoles of various concentration for 6 h. Cells were treated with MTT (Invitrogen) solution for 2 h, and then with Dimethyl sulphoxide (DMSO). The absorbance was read at 540 nm by spectrophotometer. A blank, a sample group without cells, was used as a negative control, while a sample group that was not treated with anything was used as a positive control.

### Phagocytosis assay

RAW 264.7 cells were adjusted to 5 × 10^4^/ml, cultured for 24 h and starved for 12 h. The cells were then pre‐treated with HBP vacuoles in various concentrations of (0.001, 0.005 and 0.01) μg/ml for 6 h. Cells were then treated with Zymosan suspension provided by Phagocytosis assay kit (Cell biolabs) for 2 h, and the experimental procedure was carried out according to the product manual. pVMA11 vacuoles and phagocytosis inhibitor (Cytochalasin D) were used as controls.

### Estimation of histamine concentration

Pre‐treatment to examine the immune‐stimulating effect of the HBP vacuoles was conducted. Histamine released from the stimulation of HBP vacuoles and Lipopolysaccharide (LPS) in RAW 264.7 cells was estimated using Histamine ELISA kit (Enzo life science). The experiment was conducted as described in the product manual. RAW 264.7 cells were adjusted to 5 × 10^4^/ml, cultured for 24 h and starved for 12 h. Cells were pre‐treated with HBP vacuoles for 6 h at various concentration of (1–10) ng/ml, washed twice with DPBS and then proceeded to the next step. Cells were then treated with LPS at 1 μg/ml for 24 h. The HA concentration of the supernatant was estimated using Histamine ELISA kit.

Histamine ELISA was performed to confirm that HA released from LPS stimulation of the RAW 264.7 cells (Dy et al., 1981) could be inhibited by treatment with HBP vacuoles. RAW 264.7 cells were adjusted to 5 × 10^4^/ml, cultured for 24 h and starved for 12 h. Cells were pre‐treated with LPS at 1 μg/ml for 24 h. The cell supernatant was treated with various concentration of HBP vacuoles of (1–10) ng/ml for 2 h. Then, the HA concentration of the supernatant was estimated by Histamine ELISA. pVMA11 vacuoles were used to compare the effects on the recombinant vacuoles as controls.

### Data analysis

Each data point was obtained from three independent samples conducted simultaneously for error analysis. The averages are reported with the standard error of mean and correlations for several experimental conditions. The data were analysed using SigmaPlot (Systat Software, Inc., USA).

## RESULTS AND DISCUSSION

### Histamine suppression immune enhancer, histamine‐binding protein vacuoles

In this study, we aimed to design a biocompatible material that enhances immunity, and at the same time, modulates HA. HBP vacuoles were designed using cloning technology, which can be called bioengineering. The yeast vacuoles were used as recombinant materials. In addition, HBP vacuoles were targeted to express HBP on the vacuole surface through genetic manipulation. To do so, the pVMA11 protein, which modified vacuolar membrane ATPase (VMA11) protein to direct the target protein to the cytoplasm, was used. In addition, GFP and His‐tag were positioned to check the recombinant protein expression. HA is expected to bind to HBP on the cytoplasmic side of the HBP vacuoles.

**FIGURE 1:**
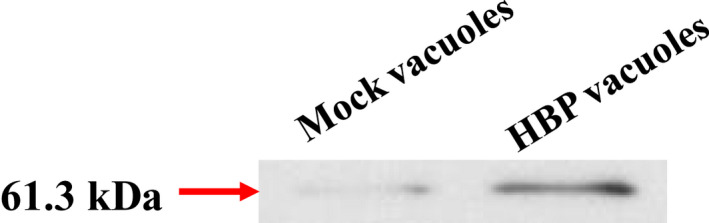
Western blot for recombinant protein. To confirm the expression of recombinant protein, western blot was performed using His‐tag. The recombinant protein was composed of signal sequence (SS), histamine‐binding protein (HBP), green fluorescence protein (GFP) and Part 2 and Part 1 of pYES2 vector. The size of the protein was 61.3 kDa. The mock vacuoles were used as a control as recombinant yeast transformed only pYES2 vector.

Gene expression in recombinant yeast was induced by galactose, and HBP vacuoles were isolated from recombinant yeast by centrifugation. As shown in Figure 1, the protein expression was confirmed by Western blot, which used ligated His‐tag. In addition, the expected size of the protein was 61.3 kDa. Mock vacuoles were used as controls to the recombinant yeast vacuoles transformed only pYES2 vector.

**FIGURE 2:**
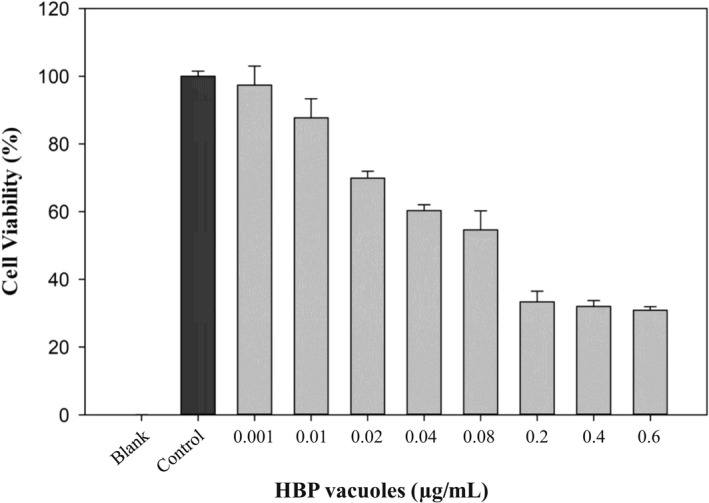
The effect of HBP vacuoles on cell viability of RAW 264.7 cells. RAW 264.7 cells were treated with HBP vacuoles for 6 h at various concentrations, and cell viability was estimated by MTT assay. As a result, HBP vacuoles of 0.01 μg/ml was the highest concentration with a cell viability of 80% or more. All experiments were performed in triplicate, and data are shown as the mean ± SEM.

MTT assay was conducted to confirm the in vitro application of HBP vacuoles, and it was performed to decide an appropriate concentration of HBP vacuoles for RAW 264.7 cells. RAW 264.7 cells were treated with HBP vacuoles by concentration, and the cell viability was calculated. As shown in Figure 2, HBP vacuole of 0.01 μg/ml was the highest concentration, with a cell viability of 80% or more. Therefore, the experiment was conducted up to 0.01 μg/ml at the concentration of the maximum HBP vacuoles.

### Stimulation of immune responses

**FIGURE 3:**
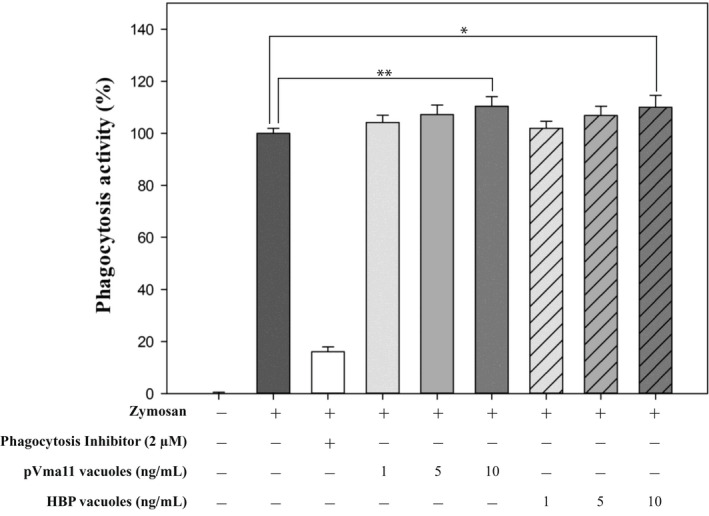
The phagocytosis activity treated HBP vacuoles. RAW 264.7 cells were treated with HBP vacuoles for 6 h, and the cells were stimulated with zymosan for 2 h. The phagocytosis activity was estimated using phagocytosis assay kit. All experiments were performed in triplicate, and data are shown as the mean ± SEM.

Phagocytosis assay was performed to determine how the substances in this study work in vitro. The phagocytosis activity according to the concentration of vacuoles was estimated by treating the yeast vacuoles in RAW 264.7 cells, which are macrophages. As shown in Figure 3, HBP vacuoles increased the phagocytosis of RAW 264.7 cells in a concentration‐dependent manner. At the maximum concentration of HBP vacuoles of 0.01 μg/ml, about 110.1% of phagocytosis was shown. The pVMA11 vacuoles, used as controls, also increased phagocytosis in a concentration‐dependent manner. At the maximum concentration of pVMA11 vacuoles of 0.01 μg/ml, about 110.4% of phagocytosis was shown. As a result of another control group, a phagocytosis inhibitor (Cytochalasin D), it was confirmed that the phagocytosis assay was well conducted, because the phagocytic activity was measured as 16.1%. From the results of pVMA11 vacuoles, it was confirmed that yeast vacuoles can enhance immunity by stimulating phagocytosis. In addition, the recombinantly expressed protein, HBP, did not interfere with the immune‐enhancing effect of the yeast vacuoles.

**FIGURE 4:**
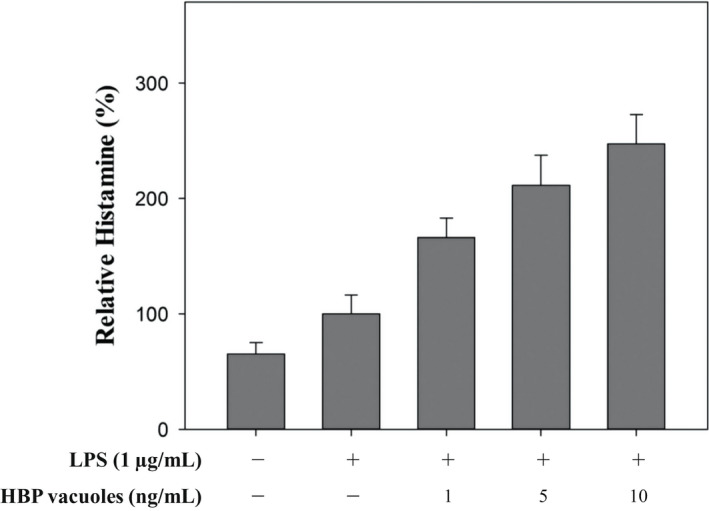
Histamine release by immune‐stimulatory responses to RAW 264.7 cells of HBP vacuoles. This experiment was conducted to confirm the effect of HBP vacuoles on the immune response to immune cells. Histamine was released from the RAW 264.7 cells, and the released histamine was measured by Histamine ELISA. RAW 264.7 cells were pre‐treated with HBP vacuoles for 6 h, washed with DPBS and reacted with LPS for 24 h. As a result, the RAW 264.7 cells released about 250% more histamine in the sample group treated with both HBP vacuoles and LPS, compared to the sample group treated with only LPS. All experiments were performed in triplicate, and data are shown as the mean ± SEM.

The amount of HA released from RAW 264.7 cells was measured to confirm the immune‐stimulatory effect on HBP vacuoles. Macrophages secrete HA when immune‐stimulated (Takamatsu et al., 1996; Takamatsu & Nakano, 1995), and the HA can induce immune‐stimulation on itself or other macrophages (Dy et al., 1981; Romero et al., 2016). HA has a very short half‐life, and the amount released varies, depending on the state of the cell (Taylor & Snyder, 1972). Therefore, the results were summarized by calculating the relative HA value while setting the positive control as 100%. Positive controls treated only with LPS were taken as 100%. As shown in Figure 4, it was confirmed that the HA released from the sample group treated with only LPS increased, compared to the non‐treated sample group. In addition, when cells were treated with LPS after being treated with HBP vacuoles, the HA was increased in a concentration‐dependent manner, and it was confirmed that HA was increased up to 250%. As a result, it was confirmed that the HBP vacuoles can stimulate the immune response of RAW 264.7 cells, which are immune cells.

### Histamine inhibition of histamine‐binding protein vacuoles

**FIGURE 5:**
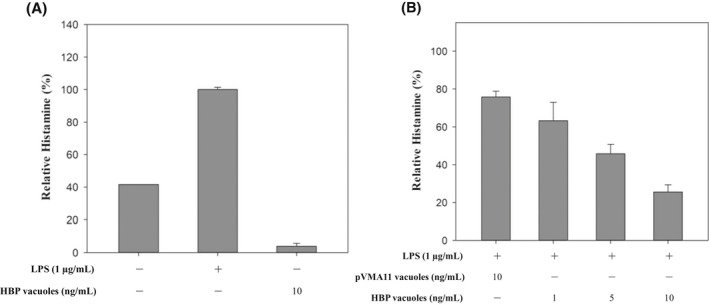
Histamine inhibitory effect of HBP vacuoles. The histamine was released by RAW 264.7 macrophage cells, and the released histamine concentration was estimated by Histamine ELISA assay. (a) RAW 264.7 cells were pre‐treated with LPS for 24 h, and then HBP vacuoles were added to the suspension. So, we confirmed whether the HBP vacuoles reacted with the histamine or not. (b) The histamine inhibitory effect was confirmed by treating the recombinant vacuoles by concentration. Compared to the control pVMA11, HBP vacuoles reduced histamine in a concentration‐dependent manner. Relative histamine was calculated by assuming 100% of the positive control result through several replicates. All experiments were performed in triplicate, and data are shown as the mean ± SEM.

The effect of inhibiting HA secreted by the immuno‐stimulation of RAW 264.7 cells was confirmed. RAW 264.7 cells were induced to release HA through immune‐stimulation and were then treated with HBP vacuoles. RAW 264.7 cells pre‐treated with LPS induced HA release. The HA concentration released from RAW 264.7 cells was estimated by Histamine ELISA. Figure 5(a) confirms the amount of HA released when treated with LPS. Figure 5(b) shows that HA was reduced by about 74.9% and decreased in a concentration‐dependent manner. This is a very significant result even compared with the control pVMA11 vacuoles. This result confirmed that the recombinantly expressed histamine‐binding protein (HBP) located on the surface of the vacuole can function to bind the HA, and that the HA inhibitory effect is significant.

## CONCLUSION

In this study, a substance to enhance weakened immunity, and to relieve side effects caused by excessive HA secretion, was developed and evaluated. The vacuole is a biocompatible organelle isolated from yeast. In addition, it can be easily obtained by culturing and isolating, enabling mass‐production (Johnson & Echavarri‐Erasun, 2011). Also, it fulfils its role even at low concentration. For these reasons, HBP vacuoles enhance immunity, and are expected to be used as an effective material for HA inhibition. However, it seems necessary to study HBP vacuoles to be applied to skin, such as skin cells and artificial skin tissues. The application to actual skin model in vivo should also be confirmed. If the above experiments are carried out, HBP vacuoles could provide a new solution of HA inhibitors that enhance immunity.

## AUTHOR CONTRIBUTIONS

H. Jang performed all of experiments and wrote the main manuscript text. Y.‐H. Kim and J. Min designed the overall experimental concept and revised the manuscript.

## FUNDING INFORMATION

Korea Institute of Planning and Evaluation for Technology in Food, Agriculture and Forestry (IPET), (Grant/Award Number: ‘321108‐04’).

## CONFLICT OF INTEREST

The authors declare no competing financial interests.

## Data Availability

The gene sequences used for yeast vacuole surface expression of HBP and VMA11 can be searched in NCBI GenBank database with the accession number U96081 and NC_001148. The strain of S. cerevisiae 2805 used for transformation and protein expression is ATCC 208280.
